# Resectable Pediatric Nonrhabdomyosarcoma Soft Tissue Sarcoma: Which Patients Benefit from Adjuvant Radiation Therapy and How Much?

**DOI:** 10.5402/2012/341408

**Published:** 2012-03-15

**Authors:** Lynn Million, Sarah S. Donaldson

**Affiliations:** Department of Radiation Oncology, Stanford Cancer Center, 875 Blake Wilbur Drive, Stanford, CA 94305, USA

## Abstract

It remains unclear which children and adolescents with resected nonrhabdomyosarcoma soft tissue sarcoma (NRSTS) benefit from radiation therapy, as well as the optimal dose, volume, and timing of radiotherapy when used with primary surgical resection. This paper reviews the sparse literature from clinical trials and retrospective studies of resected pediatric NRSTS to discern local recurrence rates in relationship to the use of radiation therapy.

## 1. Introduction

NRSTS in the pediatric age group is a challenging group of tumors to manage due to a variety of reasons, particularly for the radiation oncologist. The diversity of histologic subtypes may influence the sensitivity of tumors to radiation. The spectrum of anatomical sites may preclude a complete marginal resection in certain sites, which in turn influences the dose of radiation needed to control disease. The patterns of local tumor spread also influence the optimal treatment volume. Additionally, the uncommon occurrence of pediatric NRSTS, which is only approximately 500 cases per year, and the wide age distribution from infancy to adulthood add to the challenges of determining appropriate radiation dose, volume, and timing relative to surgical resection [1]. Many radiation oncologists reference guidelines established for adult soft tissue sarcomas for radiation therapy dose and volume based on sarcoma grade, tumor size, and resectability. However, patients in the pediatric age group are unique from adults for many reasons including growth and fertility considerations and longer life span to develop late effects of radiation including an enhanced risk for second malignant tumor induction.

Radiation therapy dose and volume guidelines for rhabdomyosarcoma (RMS), the most common soft tissue sarcoma in childhood, have been established through a series of phase III prospective trials conducted over 4 decades by the Intergroup Rhabdomyosarcoma Study Group (IRSG) and COG [2–5]. Similar trials have not been conducted for the pediatric NRSTS. As no standard of care currently exists for managing this diverse group of tumors, the current challenge for children with soft tissue sarcoma, other than rhabdomyosarcoma, is to identify which patients benefit from the addition of radiation therapy and to determine the lowest optimal radiation dose and volume to be administered, so as to avoid injury to normal tissues and minimize risk for the development of a second cancer.

## 2. Who Benefits from Adjuvant Radiation Therapy for Resectable Pediatric NRSTS?

Patients with resectable soft tissue sarcoma are known to have a superior outcome as compared with those having a tumor which is not initially resectable. Patients with a sarcoma resected at the time of diagnosis have an estimated 5-year survival rate 89% as compared to those whose tumor is unresected at diagnosis, with 5-year survival estimated to be just 60% [6, 7]. Although complete surgical resection is the cornerstone to curative therapy in pediatric NRSTS, the question for the radiation oncologist is whom of those with resectable disease will fail locally and thus might benefit from the addition of radiation therapy.

The answer to this question is complex and multi-factorial. The only United States multicenter trial for resectable pediatric NRSTS was conducted through the Pediatric Oncology Group (POG) from 1986 to 1993. Although this POG 8653 trial was designed to test the efficacy of adjuvant chemotherapy in resectable pediatric NRSTS, the radiation therapy guidelines provide insight as to the evolution of treatment recommendations. Group I patients with completed resected disease did not receive postoperative radiation. Group II patients with microscopic residual disease received postoperative radiation therapy based on their age and potential for growth. Patients younger than 6 years of age received 35 Gy followed by a field reduction to a total dose of 45 Gy. Those 6 years and older received 45 Gy followed by a field reduction to a total dose of 50 Gy. Although outcome data was not reported separately for the Group I (55 patients) and Group II (25 patients), the analysis suggests that among 80 patients, there were 8 patients with a local recurrence (12.5%) with at least 5 occurring in Group I patients who did not receive radiation; the majority of these were high grade tumors. The most important finding of this study was that POG grade 3 tumors fared much worse (52% survival at 5 years) as compared with POG grade 1 or 2 sarcomas (92% 5-year survival) defining tumor grade as an important prognostic factor influencing outcome [8, 9].

The IRSG clinical grouping classification that was used to stage patients on this trial is frequently used today. However, it has not been validated as a universally accepted or an appropriate staging system for pediatric NRSTS as it does not account for important factors, such as tumor grade and depth of clear surgical margins. For example, the width of the normal tissue from inked surface of the surgical specimen to viable tumor is not specified in this classification, only that a “cuff of normal tissue” is required to allocate patients into Group I. Group II included patients with microscopic positive margins [10].

We know from retrospective single institution studies that for completely resected tumors, the width of a clear surgical margin influences the local recurrence rate. Blakely et al. reviewed the St. Jude Children's Research Hospital experience of 88 children with NRSTS in which the IRSG classification was used in Group I patients to determine the local recurrence rate in relationship to the width of the pathologic surgical margin. Patients whose tumors revealed a surgical margin of ≥1 cm had fewer local recurrences regardless of tumor grade. Those with a surgical margin <1 cm and low-grade histology did not benefit from routine postoperative radiation, whereas patients with a high-grade tumor had improved local control with postoperative radiation therapy. Of 14 patients with a <1 cm surgical margin and high grade tumors, 7 had postoperative radiotherapy, and no recurrences were observed, whereas of the 7 patients who did not receive postoperative radiotherapy, 3 (42.9%) suffered a local recurrence. Of 20 patients with at least a 1 cm negative surgical margin and a high grade tumor, none received postoperative RT and 4 recurred (20%). This report suggested that radiation therapy most benefits those with less than a 1 cm margin and those with a high-grade tumor [11].

Spunt et al. extended the St. Jude Children's Research Hospital experience assessing prognostic factors for pediatric patients presenting with resectable NRSTS and analyzed 121 patients treated between 1969–1996. Of 81 Group I patients, 61 had at least 1 cm surgical margin, whereas 20 had less than 1 cm margin. Only 10 patients received adjuvant radiation therapy with a median dose of 54.9 Gy (range 26.5 to 60.4 Gy). Univariate analysis showed that the width of the margin did not influence local recurrence nor did the addition of postoperative radiation for the small group (10%) who received radiation therapy. However, almost 60% of the Group I patients had low-grade (POG grade 1and 2) tumors. Of 40 Group II patients, 21 patients received radiation therapy with a higher median dose of 59.4 Gy (range of 40 to 75 Gy). The 5-year estimated cumulative incidence of local failure was 12.8% for Group I and II patients. 17 of 121 patients had a local recurrence only. Two patients relapsed with both local and distant disease. Factors associated with local recurrence were microscopic residual disease, large tumor size, and intra-abdominal disease, whereas improved local control was associated with the use of radiation therapy. Of note, these authors report that of 76 low-grade tumors, 10 recurred (13%), but 9 patients were alive with retreatment with only one patient death, from a radiation-induced osteosarcoma [7].

Although this retrospective review suggested that margin width for patients with Group I tumors did not influence outcome, the authors attributed this to the relatively small percentage of high-grade tumors in this cohort and a large proportion having at least a 1 cm negative surgical margin. For those with a Group II tumor, the administration of radiation reduced the local recurrence rate. It is important to note that the median radiation dose was 59.4 Gy, higher than used on the POG 8653 trial (45–50 Gy).

Smith et al. reported the outcome of 95 resectable NRSTS pediatric and young adult patients treated at the University of Florida over a 34-year period. All patients in this series received radiation therapy, either preoperatively (median dose 50.4 Gy, range 27–69.6 Gy) or postoperatively (median dose of 61 Gy, range 37.5–74.4 Gy). Although there is not a comparative cohort who did not receive radiotherapy, there are several interesting lessons from this experience. The Florida investigators used ≥1 cm as their institutional definition of a negative margin versus <1 cm as a close margin and considered microscopic residual disease as a positive margin. They reported an overall local failure rate of just 12% at 5 years. Patients with a negative margin had a statistically significant higher local control rate at 5 years by univariate and multivariate analyses with only 4 of 64 (6%) local recurrences observed versus 8 of 30 (27%) when the margin was close or positive. The authors did not observe an association of grade with local recurrence; however they had a small number of patients with a low-grade tumor who received radiotherapy, reflecting the institutional policy of carefully selecting patients in whom radiation therapy might be avoided. Of note, the authors reported that all patients who suffered a local failure ultimately died of disease [12].

None of these series are readily comparable, but taken together they suggest that for patients with a completely resected tumor, defining the width of a negative surgical margin is an important factor in determining who will benefit from the addition of radiation therapy. In the pediatric and young adult population it is often challenging to achieve a 1 cm surgical margin, simply due to the relative lack of tissue as compared with an adult. Additionally, certain primary sites, such as the head and neck and retroperitoneal region, may be very difficult anatomic regions to achieve a clear margin. Therefore, lesser margins are often accepted in the pediatric and young adult age group, as compared with adults. The current Children's Oncology Group (COG) NRSTS trial for pediatric and young adults is prospectively testing whether 0.5 cm or greater in all directions around the tumor and/or tumor abutting the fascia or periosteum that is removed in continuity with the tumor specimen is an appropriate negative surgical margin.

## 3. Other Prognostic Factors to Consider for the Use of Adjuvant Radiation Therapy

Histologic grade of tumor also has prognostic value for determining if the addition of radiation therapy may benefit those with a higher risk for local relapse. Two grading systems commonly used in pediatric soft tissue sarcomas are a POG grading system and the French Federation of Cancer Centers (FNCLCC) grading system. The POG system is based on histologic type, necrosis, and mitosis, whereas the French Federation of Cancer Centers (FNCLCC) grading system is based on differentiation, necrosis, and mitosis [9, 13, 14].

Whether one grading system is superior for predicting outcome is unknown. However, it is important to know which grading system is used when reviewing retrospective series with regard to local recurrence and distant metastases. For example, in Spunt's retrospective analysis of prognostic factors in pediatric NRSTS, POG grade 1 and 2 tumors were combined into a low grade group and compared with POG grade 3 high-grade tumors. This subdivision is supported by the results of the prospective POG 8653 trial analysis which showed similar superior outcomes for the POG grade 1 and 2 tumors as compared with poorer outcome for high grade—POG grade 3 tumors. In Spunt's series, no patients with a low-grade tumor died of disease despite withholding post-operative radiation therapy for those patients with a close or a positive margin (Group I or Group II). Taken together these data support including intermediate POG grade 2 into a lower grade category [7]. The importance of this distinction is that distant metastases are rare in patients with a low-grade sarcoma. Therefore local recurrence after surgery is rarely life threatening. A management strategy of careful observation in those presenting with a low-grade tumor with close and/or positive margin is often adopted in an attempt to avoid or delay the administration of radiation therapy. Reexcision with possible radiation at the time of recurrence can result in an excellent overall survival. An exception to this strategy would be if the location of the primary tumor would require an amputation or other mutilating procedure if the tumor does recur locally.

In distinction to the low-grade tumors, resectable high-grade tumors carry an increased risk for local recurrence and distant metastases. As noted in the above studies, patients with a Group II tumor who have a high-grade sarcoma are shown to have a decreased risk for local recurrence when adjuvant radiation therapy is administered. Whether optimizing local control improves overall survival remains unclear, with some series suggesting a survival benefit when the local recurrence rate is low [7, 11, 12, 15, 16].

For patients with a Group I high grade sarcoma, in addition to the margin width, tumor size and invasiveness also influence outcome. Ocku et al. reported an association of unfavorable outcome in those with a resectable synovial sarcoma with invasiveness and large size (>5 cm). In this multicenter, multivariate analysis of synovial sarcomas in childhood and adolescence, the results of four large research groups were analyzed. Both tumor size (>5 cm) and invasive disease (invading contiguous tissue or organs) were prognostically significant for poor event free survival (EFS) and overall survival (OS) in those patients with a Group I or II disease. Of these Group I and II patients, only univariate analysis confirmed an association with an invasive tumor and a higher risk of local recurrence. When radiation therapy was delivered, the median dose was 50 Gy with a range from 16 to 68 Gy. When the impact of radiation therapy on local recurrence free survival was evaluated, there was not a statistically significant local recurrence free survival benefit whether patients received radiation therapy (96% at 5 years) compared to those in whom it was omitted (87% at 5 years). The authors hypothesize that the small number of local recurrences or possible misclassification of Group I and II patients could contribute to the noted lack of benefit from radiation therapy [15].

The analysis by Orbach et al. provides an opportunity to review the results of a treatment strategy designed to avoid radiation therapy in young patients with localized synovial sarcoma. Pooling three prospective trials on pediatric patients with synovial sarcoma conducted from 1984 to 2003 through the International Society of Pediatric Oncology Malignant Mesenchymal Tumor study (SIOP-MMT), there were 21 IRS Group I patients with synovial sarcoma. Only 2 patients received postoperative radiotherapy, with a dose of 45 Gy recommended on all 3 trials. There were 3 local recurrences (15%), all occurring in patients who did not receive radiation, and all were alive without disease at the study report. The 2 patients who received radiotherapy had a metastatic relapse and both died of disease. Although the 5-year EFS was 69% for Group I patients, the OS at 5 years was 90%, leading the authors to conclude that radiation therapy could be omitted in this population of patients. This series suggests that for completely resected synovial sarcomas, local failure does not portend a worse outcome as retreatment may be successful [17].

Of note, synovial sarcomas are generally regarded as high-grade sarcomas; however, this remains an unresolved pathologic controversy. If a tumor has some intermediate grade features, a more favorable outcome might be expected which may influence the recommendation regarding adjuvant therapy [18]. Ladanyi et al. report that the molecular signature of the SYT-SSX fusion type provides the strongest prognostic information for localized synovial sarcoma compared with all other prognostic factors including histologic grade, age, sex, and anatomic location [19]. Pathologic and molecular research will continue to enhance our knowledge of appropriate therapy when there may be a discrepancy between morphology and molecular analysis.

Another common NRSTS in the pediatric and young adolescent age group is malignant peripheral nerve sheath tumor (MPNST). These tumors have a predilection for local recurrence and distant metastases with poorer OS than that seen in synovial sarcoma. Patients with neurofibromatosis 1 (NF1) who develop MPNST have a poorer prognosis as compared with patients without NF1. Carli et al. reported the results of patients with MPNST treated in Italian and German studies between 1975 and 1998. The analysis showed progression free survival (PFS), and OS for Group I was 61% and 82%, whereas Group II was 37% and 62%, respectively. 25% of Group I patients received adjuvant radiation therapy with a local recurrence rate of just 17%. For those Group I patients in whom radiation therapy was omitted, the local recurrence rate was 36%. For Group II patients, the local recurrence rate with radiation therapy was 45% versus 68% without radiation therapy. The radiation dose in this study population was 45 Gy using a hyperfractionated treatment with 1.6 Gy given twice daily [20]. This series suggests that local control is a critical problem, even for completely resected disease suggesting an inherent biologic resistance to current therapy.

Many soft tissue sarcomas in the pediatric and young adult age group are unable to be histologically subtyped with current immunohistochemistry stains and sophisticated molecular testing and are hence labeled as unclassified or undifferentiated NRSTS. The IRSG trials included undifferentiated sarcomas in their studies. Although undifferentiated sarcoma accounted for a small proportion (4%) of the total eligible patients on IRS IV, the 3-year EFS was statistically inferior (55%) when compared with similar therapy for alveolar RMS (66%) and embryonal RMS (83%). On this trial, Group I patients did not receive radiation therapy unless they had pretreatment poor prognostic features (unfavorable site and either clinical evidence of regional node involvement or an invasive tumor), in which case they received 41.4 Gy. All Group II patients received 41.4 Gy [4]. In current RMS trials conducted through COG soft tissue sarcoma committee, undifferentiated or unclassified sarcomas are ineligible histologic subtypes for study entry, but are included as a histologic subtype eligible for a NRSTS trial.

Age may influence the decision to recommend adjuvant radiation therapy. Hayes-Jordan et al. reported young age as a favorable prognostic factor for pediatric NRSTS. Improved outcome was attributed to younger patients having smaller, lower-grade tumors with less invasive characteristics, such as myxofibrosarcoma, with no patients less than 1 year of age with metastatic disease at diagnosis. Comparatively, older patients (>15 years of age) were more likely to have higher grade, more invasive sarcomas, such as synovial sarcoma and MPNST, and more advanced disease at diagnosis [21]. Even within the same histologic subtype older age appears to remain a poor prognostic factor. Okcu et al. in their series on synovial sarcoma report this effect of age, with younger patients faring better than those diagnosed at an older age [15]. Age may possibly be used as a factor for withholding radiation or reducing the radiation dose in certain youngsters presenting with a resectable high-grade tumor where the latent long-term risks associated with the addition of radiation therapy may outweigh the benefit of such therapy.

An additional concern when considering the administration of radiation therapy may be the predilection of the patient to develop a second cancer as a result of carrying an underlying germline mutation where ionizing radiation may upregulate critical molecular pathways associated with tumorigenesis. One of the more common non-rhabdomyosarcomas in the pediatric age group, MPNST, has been associated with underlying germline mutation in NF1 in about 50% of patients. An array of NRSTS tumors are associated with a germline mutation such as P53 (as observed in patients with the Li-Fraumeni syndrome) where exposure to radiation is associated with an increased risk for causing a latent secondary cancer [22].

## 4. Radiation Therapy Dose, Volume, and Treatment Techniques

Despite the attendant risks associated with treatment, some patients with resectable pediatric and young adult NRSTS benefit from the administration of radiation. As the desired goal of radiation therapy is to optimize local control with a minimal effect on quality of life, several different strategies may be considered. Patients with subclinical disease may not require the same radiation dose as those who clearly have microscopic residual disease. Modifying the radiation dose according to extent of disease is a concept well known to radiation oncologists since Fletcher proposed a graduated dose scheme for adults with head and neck carcinoma to sterilize subclinical lymph nodes versus a higher dose to treat a primary site with microscopic or gross disease. Doses of 50 Gy were recommended for subclinical disease, 60 Gy for microscopic disease, and 70 Gy for gross disease [23]. Using a similar paradigm, it may be feasible to use a lower dose in the setting of a clearly negative margin, such as 45–50 Gy, and a slightly higher dose of 50–55 Gy for a true microscopically involved margin.

In addition, the POG 8653 trial, which adjusted the radiation dose according to age, is supported by data suggesting younger children far better than older adolescents, even within the same histologic subtype, such as seen in synovial sarcoma.

Better understanding of which sarcomas are more likely to be radiosensitive, through analysis of the pathologic treatment effect in patients who have received preoperative radiation therapy, may allow dose adjustment. For example, Roberge et al. reported on the imaging and pathologic response of radiation therapy for extremity and truncal soft tissue sarcomas in 50 patients. This report showed an association between reduction in tumor volume by imaging predictive of a pathologic treatment response in the surgical specimen. Some histologic subtypes were shown to be very radiation therapy sensitive, such as myxoid liposarcoma, where a substantial decrease in tumor volume (82.1%) on imaging was associated with a high percent of tumor necrosis in the pathologic specimen. High-grade sarcoma which showed very minimal reduction (<1%) in the tumor volume by imaging was associated with minimal treatment-related effect seen in the pathologic specimen.

Other strategies to minimize late effects of treatment might be to consider the use of preoperative radiation therapy in those patients presenting with a large, invasive, and high-grade tumor that would likely require radiation therapy based on the pretreatment evaluation [24]. Preoperative radiation has the potential advantage of treating a smaller volume with lower doses potentially resulting in fewer complications. A Canadian sarcoma randomized trial reported fewer long-term complications in patients who received preoperative radiation therapy as compared with postoperative therapy [25].

One of the most effective ways to minimize the late effects of radiation therapy is to design the radiation field to protect the adjacent normal tissues. The best way to accomplish this is to use smaller treatment margins around the tumor volume. Historically, wide margins up to 5 cm or greater were used to treat potential subclinical disease far beyond the operative bed or radiographic findings. In turn, such a large volume would invariably include critical organs and normal tissues vital for form and function.

Krasin et al. prospectively tested whether a smaller margin on the target volume could be used and not compromise local control. Of 32 pediatric and young adults with a high-grade NRSTS, 27 received adjuvant radiation therapy. Using a 2 cm margin around the tumor volume at diagnosis and delivering a median cumulative postoperative radiation dose of 60 Gy (range 41.4–70.4 Gy) or a preoperative dose of 45 Gy (range 45–50.4 Gy), the 3-year cumulative local recurrence rate for patients who underwent a marginal or complete resection was <4%. There were no failures in the patients with clear surgical margins. Those who failed locally did so within the high-dose radiation volume, suggesting limited margin radiation therapy is an effective strategy to employ in pediatric patients with NRSTS. Techniques such as these would be expected to confer less normal tissue injury including a possible reduction in secondary cancer induction [26].

A current COG trial for pediatric and young adults with NRSTS uses a risk-based stratification algorithm tailoring radiation therapy dose and volume based on tumor grade, size, and margin status. The results of this trial will hopefully provide critical answers to whom benefits from adjuvant radiation therapy.

In order to optimize the delivery of radiation therapy, immobilization and careful delineation of the target volume to treat are critical to the successful outcome. The standard of care today is to include 3 dimensional (3D) volumetric planning using cross-sectional imaging by either computed tomographic (CT) or magnetic resonance imaging (MRI). Effective immobilization techniques provide the ability to deliver extremely conformal treatment. Additionally, sarcomas located in the chest or abdomen may shift with diaphragmatic motion during radiation therapy. Accounting for respiratory movement is now possible to allow for further refinements in the concise delivery of radiation to the target volume.

Choosing the appropriate preoperative radiographic imaging study to fuse with a treatment planning CT/MRI scan is critical, particularly in the postoperative setting where anatomy of normal structures has returned to normal position. Furthermore treatment planning systems can format preoperative images to conform to the patient's new treatment position allowing better recreation of the precise location of the original tumor in relation to normal tissues and organs.

Delivery techniques, such as intensity modulated radiation therapy (IMRT) allow sculpting the radiation dose around vital structures. Although IMRT may deliver a higher integral dose than observed using a conformal 3-D plan, the shaping and deposition of radiation therapy is more precise [27]. Particle therapy, such a proton beam therapy, has been used in pediatric patients at several centers around the world with reported results showing excellent dosimetric delineation of target with minimal scatter to normal tissues and little exit dose. The expectation is that there will be fewer late effects [28]. Brachytherapy is another excellent tool to deliver conformal high dose to target volume. Although wound complications are reported to be higher in the implanted site using brachytherapy for sarcoma therapy, this treatment is a potentially daily precision radiation therapy delivery, and conebeam computed tomography (CBCT) helps ensure that normal tissues are within millimeters of the original simulated position. All these contemporary technologies contribute to keeping the radiation therapy dose as low as possible and hence minimizing normal tissue exposure.

## 5. Conclusion

The recommendation for the administration of radiation therapy for pediatric and young adults with resected NRSTS depends on the width of negative margin, tumor grade, tumor size, invasiveness, histologic subtype, age, and underlying genetic conditions such as Li-Fraumeni syndrome. Most patients presenting with a low-grade tumor can be observed with careful physical examination and surveillance cross-sectional imaging. Clinical trials are needed to define which of the patients with a completely resected high-grade tumor may benefit from the use of radiation therapy in addition to surgical resection. The appropriate radiation therapy dose may be driven by several factors including age, extent of subclinical disease versus overt microscopic disease, tumor histology, size, and invasiveness. Limited volume radiation using emerging technology to precisely target a tumor will reduce exposure to normal tissues.
